# Antimicrobial Peptide CATH-2 Attenuates Avian Pathogenic *E. coli*-Induced Inflammatory Response via NF-κB/NLRP3/MAPK Pathway and Lysosomal Dysfunction in Macrophages

**DOI:** 10.3390/ijms252312572

**Published:** 2024-11-22

**Authors:** Yating Xu, Liuyi Xu, Tingting Zhang, Hongliang Tian, Yi Lu, Sha Jiang, Xuefeng Cao, Zhiwei Li, Xiaoxiang Hu, Rendong Fang, Lianci Peng

**Affiliations:** 1 Joint International Research Laboratory of Animal Health and Animal Food Safety, College of Veterinary Medicine, Southwest University, Chongqing 400715, China; xyt980505@email.swu.edu.cn (Y.X.); nanyouxuan@email.swu.edu.cn (L.X.); luyi2907@163.com (Y.L.);; 2 National Center of Technology Innovation for Pigs, Chongqing 402460, China; 3 Division of Infectious Diseases and Geographic Medicine, Department of Medicine, Stanford University, Stanford, CA 94305, USA

**Keywords:** cathelicidin-2, avian pathogenic *E. coli*, anti-inflammatory response, NF-κB/NLRP3/MAPK pathway

## Abstract

Cathelicidins have anti-inflammatory activity and chicken cathelicidin-2 (CATH-2) has shown to modulate immune response, but the underlying mechanism of its anti-inflammation is still unclear. Therefore, in this study, we investigated the anti-inflammatory activity of CATH-2 on murine peritoneal macrophages during avian pathogenic *E. coli* (APEC) infection. The results showed that CATH-2 priming significantly reduced the production of IL-1β, IL-6, IL-1α, and IL-12. In addition, CATH-2 significantly attenuated APEC-induced caspase-1 activation and the formation of an adaptor (ASC) of NLRP3 inflammasome, indicating that CATH-2 inhibits APEC-induced NLRP3 inflammasome activation. Furthermore, CATH-2 remarkably inhibited NF-κB and MAPK signaling pathways activation. Moreover, CATH-2 significantly inhibited mRNA expression of cathepsin B and inhibited lysosomal acidification, demonstrating that CATH-2 disrupts lysosomal function. In addition, promoting lysosomal acidification using ML-SA1 hampered the anti-inflammatory effect of CATH-2 on APEC-infected cells. In conclusion, our study reveals that CATH-2 inhibits APEC-induced inflammation via the NF-κB/NLRP3/MAPK pathway through the dysfunction of lysosome.

## 1. Introduction

Avian pathogenic Escherichia coli (APEC) infection causes colibacillosis characterized by omphalitis, salpingitis, cellulitis, and peritonitis, leading to high mortality and morbidity, resulting in big economic losses in the poultry industry [1]. The main prevalent serotypes of APEC in the world are O1, O2, and O78 associated with avian colibacillosis, which interact with host cells and lead to severe inflammation, resulting in tissue damage [2]. In addition, it has been reported that APEC and extraintestinal pathogenic Escherichia coli (ExPEC) share common virulence factors, and APEC can spread from poultry and their products to humans, which pose a potential threat to human health [3]. Due to the diversity of APEC serotypes, current commercially available vaccines fail to provide full production against all the serotypes. Antibiotics play an important role in the treatment of APEC infection, but the increase in antibiotic resistance poses a great challenge to the treatment. Therefore, there is an urgent need to search for new therapeutics against APEC infection.

APEC exerts its pathogenicity via a variety of virulence factors, such as adhesins, invasins, protectins, iron acquisition systems, toxins, etc. These factors contribute to the different processes of APEC infection, including attachment to host cells, invasion of the host cells, and causing cell damage [4]. To defend bacterial invasion, innate immune response plays an important role, but virulence factors in turn drive the harmful inflammatory response to the host. Macrophages are one of the immune cells used to produce a high level of inflammatory cytokines, including IL-1β, IL-1α, IL-6, and TNF-α [5,6], which are triggered by multiple signaling pathways, such as NF-κB, MAPK, and NLRP3 inflammasome.

Cathelicidins belonging to antimicrobial peptides (AMPs) are produced by all complex animals, which are considered to develop as alternatives or adjuvants to antibiotics not only limited to their specific killing mechanisms but also their immunomodulatory activities [7]. Cathelicidins are mature peptides cleaved from prepropeptides containing N-terminal signal peptide, a cathelin-like domain, and the carboxy-terminal mature peptide. Most catheclidins are α-helical peptides but exert different biological functions. The human cathelicidin LL-37 has shown multiple functions beyond its antimicrobial activity to provide anti-infective modulations for the host, such as direct chemoattractant activity, wound healing, anti-apoptotic and anti-inflammatory activity, etc. [8]. So far, human cathelicidin LL-37 has been developed as a therapeutic to venous leg ulcers, indicating the potential therapeutic applications of cathelicidins [7]. Other animals’ cathelicidins, such as chicken cathelicidins, have also been studied for several years. Except for the broad spectrum antimicrobial activity, chicken CATH-2 has shown similar functions to LL-37. It has been reported that CATH-2 inhibits *E. coli*-induced TLR2 and TLR4 activation via direct killing and binding to LPS [9], leading to the reduction in inflammation. Our recent study has shown that CATH-2 as second signaling promotes NLRP3 inflammasome activation in *E. coli* LPS-primed murine macrophages [10]. In addition, the immunomodulatory activity of CATH-2 has been extensively investigated in different species, such as human peripheral blood mononuclear cells (PBMCs), chicken macrophages, murine macrophages, and porcine macrophages [11,12,13,14], indicating that CATH-2 performs its activity in a species-independent manner. However, in addition to directly blocking LPS activation in the cell surface, whether CATH-2 exhibits its anti-inflammatory activity via the intracellular signaling pathway is still unknown.

In this study, we investigated the anti-inflammatory response mechanism of CATH-2 on murine peritoneal macrophages primed by CATH-2 followed by APEC infection. The results showed that primed macrophages by CATH-2 produced a lower level of inflammatory cytokines, including IL-1β, IL-1α, and IL-6 compared to non-primed cells in response to APEC infection. Furthermore, CATH-2 inhibited the APEC-induced activation of the NF-κB, MAPK, and NLRP3 inflammasome signaling pathways in CATH-2-primed cells. In addition, lysosomal function was disrupted in CATH-2-primed cells in response to APEC. Our study provides valuable information on the anti-inflammatory effect of cathelicidin against microbial infection.

## 2. Results

### 2.1. CATH-2 Inhibits APEC-Induced Transcription and Production of Inflammatory Cytokines in Macrophages

**Figure 1:**
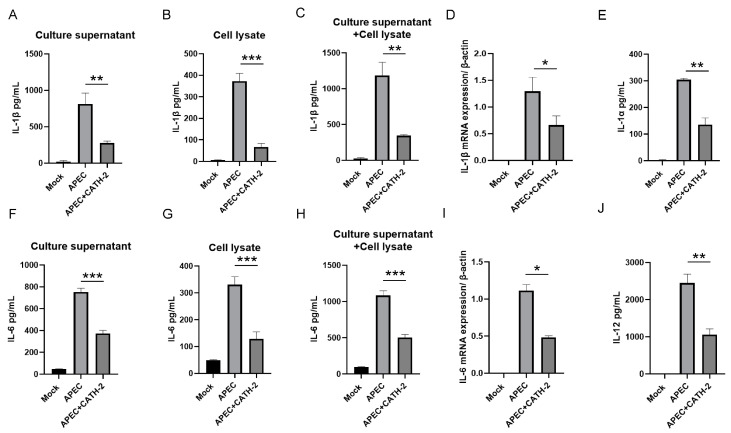
CATH-2 inhibits APEC-induced transcription and production of inflammatory cytokines in macrophages. Cells were pretreated with CATH-2 (2.5 μM) for 6 h and then infected with APEC at an MOI of 5 for 1 h. Then, gentamicin (100 μg/mL) was added to kill extracellular bacteria for the indicated time. After 24 h of infection, the level of cytokines including IL-1β (**A**–**C**), IL-6 (**F**–**H**), IL-1α (**E**), and IL-12 (**J**) in the supernatants was detected; after 6 h of infection, the mRNA expression of IL-1β (**D**) and IL-6 (**I**) was determined. Gene expression was represented via gene normalization against β-actin expression. *p* value was used to analyze significance. * *p* ≤ 0.05, ** *p* ≤ 0.01, *** *p* ≤ 0.001.

To investigate the anti-inflammatory effect of CATH-2 on APEC-infected mice primary peritoneal macrophages and to avoid the direct interaction of CATH-2 and APEC, cells were pretreated with CATH-2 for 6 h and then followed by APEC infection. After infection, cell supernatants and lysates were collected to detect the level of inflammatory cytokines. We found that CATH-2 significantly reduced APEC-induced IL-1β production in cell supernatants and lysates (Figure 1A–C). In line with IL-1β secretion, CATH-2 also significantly downregulated APEC-induced IL-1β mRNA expression (Figure 1D). Similarly, CATH-2 remarkably decreased APEC-induced secretion and mRNA expression of IL-6 (Figure 1F–I), as well as the secretion of IL-1α (Figure 1E) and IL-12 (Figure 1J). Moreover, CATH-2 did not affect cell viability (Supplementary Figure S1). These results indicate that CATH-2 could directly exert its anti-inflammatory activity on the host cells instead of only interaction with bacteria.

### 2.2. CATH-2 Inhibits APEC-Induced NLRP3 Inflammasome Activation in Macrophages

**Figure 2:**
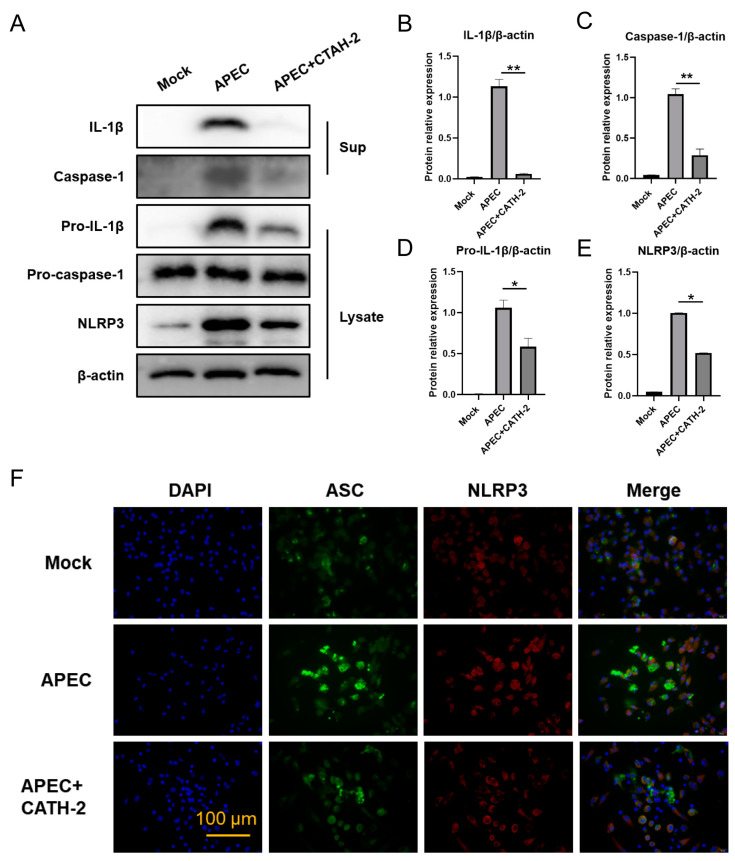
CATH-2 inhibits APEC-induced NLRP3 inflammasome activation in macrophages. Cells were pretreated with CATH-2 (2.5 μM) for 6 h and then infected with APEC at an MOI of 5 for 1 h. Then, gentamicin (100 μg/mL) was added to kill extracellular bacteria for the indicated time. After 24 h of infection, the cell supernatants and lysates were collected. Protein expression including IL-1β, caspase-1, pro-IL-1β, pro-caspase-1, and NLRP3 (**A**) was detected. Image J (1.52 a) was used to quantify the ratio of IL-1β (**B**), caspase-1 (**C**), pro-IL-1β (**D**), and NLRP3 (**E**) to β-actin. Representative images of NLRP3 (red), ASC specks (green) and cell nuclei (blue) are shown by immunofluorescent staining (**F**). *p* value was used to analyze significance. * *p* ≤ 0.05, ** *p* ≤ 0.01.

IL-1β is the key indicator of NLRP3 inflammasome activation, which is required for the recruitment of an adaptor protein called the apoptosis-associated speck-like protein containing a caspase activation and recruitment domain (ASC) to activate the caspase-1 process pro-IL-1β into biologically active IL-1β [15]. Therefore, to investigate whether decreased IL-1β secretion induced by CATH-2 is dependent on the NLRP3 inflammasome pathway, we explored the activation of the NLRP3 signaling pathway. Unsurprisingly, we found that CATH-2 significantly inhibited APEC-induced IL-1β secretion (Figure 2A,B) and the protein expression of pro-IL-1β (Figure 2D) and NLRP3 (Figure 2E). In addition, CATH-2 significantly attenuated APEC-induced caspape-1 activation while it did not affect pro-caspase-1 expression (Figure 2A). The immunofluorescent results showed that APEC induced the formation of ASC specks, while CATH-2 inhibited this effect (Figure 2F). These results demonstrate that CATH-2 inhibits APEC-induced IL-1β secretion via the NLRP3 inflammasome signaling pathway.

### 2.3. CATH-2 Inhibits APEC-Induced NF-κB and MAPK Signaling Pathway Activation in Macrophages

**Figure 3:**
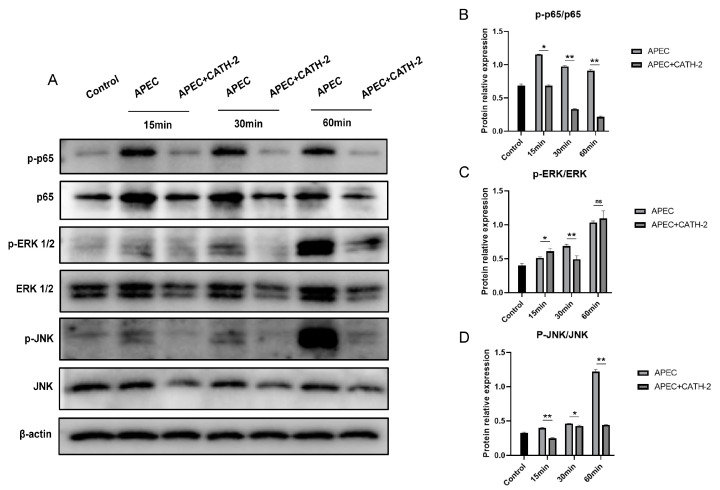
CATH-2 inhibits APEC-induced NF-κB and MAPK signaling pathway activation in macrophages. Cells were pretreated with CATH-2 (2.5 μM) for 6 h and then infected with APEC at an MOI of 5 for 1 h. Then, gentamicin (100 μg/mL) was added to kill extracellular bacteria for the indicated time. After infection, cell lysates were collected to detect NF-κB and MAPK signaling pathway activation (**A**). ImageJ was used to quantify the ratio of *p*-p65/p65 (**B**), *p*-ERK1/2/ERK1/2 (**C**), and *p*-JNK/JNK (**D**). *p* value was used to analyze significance. * *p* ≤ 0.05, ** *p* ≤ 0.01, ns = no significance.

The transcription regulator NF-κB plays an important role in the gene expression of cytokines [16]. Mitogen-activated protein kinases (MAPKs) including ERK1/2, JNK, and p38 regulate the induction of pro-inflammatory mediators, and the phosphorylation of MAPKs under the stimulation of pathogens induces the secretion of inflammatory mediators. Therefore, we investigated the effect of CATH-2 on NF-κB and MAPK signaling pathway activation. The results showed that APEC significantly increased the protein expression ratio of p-p65 to p65 at 15, 30, and 60 min post-infection while CATH-2 significantly inhibited the phosphorylation of p65 and p65 expression (Figure 3A,B), indicating that CATH-2 inhibited APEC-induced NF-κB activation. Furthermore, CATH-2 significantly downregulated the APEC-induced phosphorylation of ERK1/2 and JNK as well as the expression of ERK1/2 and JNK (Figure 3C,D), indicating that CATH-2 inhibited APEC-induced MAPK activation. Our results demonstrate that the APEC-induced inflammatory response involved in the NF-κB/MAPK signaling pathway could be inhibited by CATH-2.

### 2.4. CATH-2 Inhibits APEC-Induced IL-1β Secretion by Promoting Lysosomal Dysfunction in Macrophages

**Figure 4:**
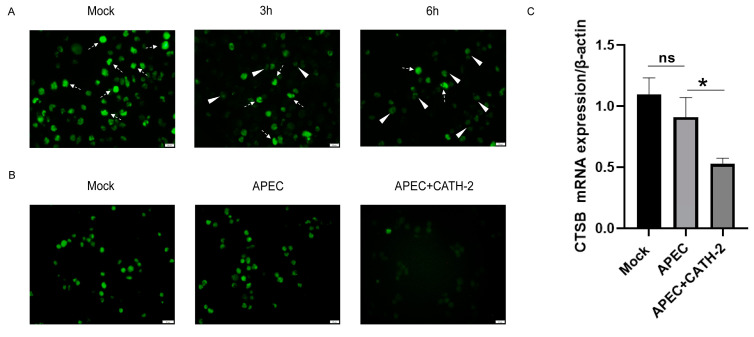
CATH-2 disrupts lysosomal integrity and acidification in APEC-infected macrophages. Cells were loaded with Alexa 488 Dextran for 1 h and then treated with CATH-2 (2.5 μM) followed by bacterial infection for a further 3 and 6 h. Representative images are shown. Dashed white arrows indicate fluorescent signal in lysosomes and solid white arrows indicate lysosomal leakage (**A**). Scale bar, 20 μm. After 2 h of infection, LysoSensor Green was used to detect lysosomal acidification (**B**). Scale bar, 20 μm. After 6 h of infection, CTSB mRNA expression was determined (**C**). *p* value was used to analyze significance. * *p* ≤ 0.05, ns = no significance.

Lysosomal function, such as lysosomal integrity and acidification, plays a critical role in the degradation of invading pathogens. In this study, we further investigated the effect of CATH-2 on lysosomal function. Firstly, fluorescent-labeled Dextran and LysoSensor Green DND-189 was loaded in the cells to detect the integrity of lysosomal function. The results showed that Dextran induced the accumulation of fluorescent signal in lysosomes (Figure 4A, white dashed arrows), but CATH-2 promoted the diffusion of the fluorescent signal (Figure 4A, white solid arrows), demonstrating that CATH-2 promotes lysosomal leakage. Furthermore, CATH-2 significantly inhibited lysosomal acidification with the weak fluorescence intensity of LysoSensor Green DND-189 (Figure 4B). Cathepsin B (CTSB) is a lysosomal encapsulated cellular protease with endopeptidase activity, which can drive protein hydrolysis and degradation in lysosomes. Our results showed that CATH-2 pretreatment significantly inhibited the mRNA transcription of CTSB (Figure 4C) at 6 h post-infection. These results indicate that CATH-2 induces lysosomal dysfunction via promoting lysosomal leakage and inhibiting lysosomal acidification.

**Figure 5:**
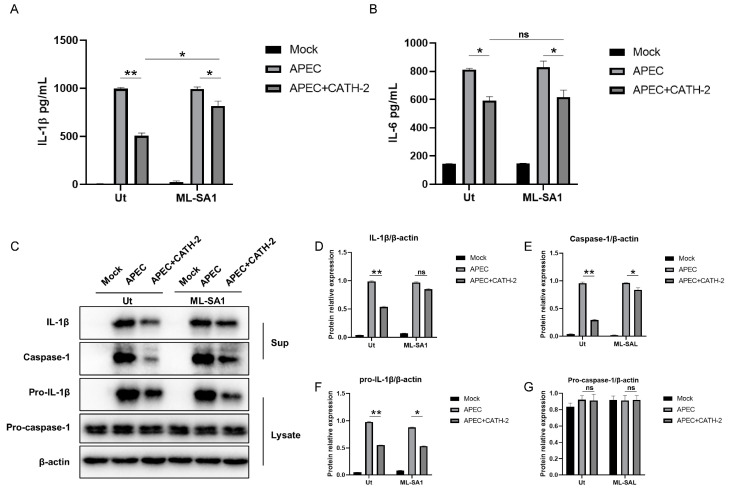
CATH-2 inhibits APEC-induced IL-1β secretion by promoting lysosomal dysfunction. Cells were pretreated with ML-SA1 for 24 h and then treated with CATH-2 treatment (2.5 μM) followed by APEC infection. The secretion level of IL-1β (**A**) and IL-6 (**B**) was determined by ELISA. Western blot (**C**) was used to detect protein expression of IL-1β (**D**), caspase-1 (**E**), pro-IL-1β, and (**F**) caspase-1 (**G**). *p* value was used to analyze significance. * *p* ≤ 0.05, ** *p* ≤ 0.01, ns = no significance.

To investigate the effect of lysosomal protease activity on the anti-inflammatory effect of CATH-2, the acidification accelerator ML-SA1 was used to restore the function of lysosome. The results showed that ML-SA1 weakens the inhibitory effect of CATH-2 on IL-1β production (Figure 5A), but IL-6 production was not affected (Figure 5B). In addition, ML-SA1 attenuated the inhibitory effect of CATH-2 on caspase-1 activation (Figure 5C,E), but CATH-2 did not affect the expression of pro-caspase-1 and pro-IL-1β (Figure 5C,F,G). These results suggest that CATH-2 inhibits the inflammatory process by disrupting lysosomal function.

## 3. Discussion

Antibiotic resistance limits the treatment efficacy to avian colibacillosis in chickens, turkeys, and other poultry. Therefore, it is urgent to develop new anti-infective drugs for alternatives or adjuvants to antibiotics. Undoubtably, the efficient killing of bacteria is one of the most useful strategies to develop new anti-infective agents. Of note, residual toxins in vivo also cause severe inflammation and damage organs, so the anti-inflammatory activity of antibacterial agents is the key to treating microbial infection. Antimicrobial peptides (AMPs) have broad spectrum antibacterial activity and immunomodulatory activity, meaning they are potential alternatives to antibiotics. It has been reported that chicken CATH-2 exhibits excellent antibacterial activity against *E. coli*, *Staphylococcus aureus* (*S. aureus*), *Streptococcus suis* (*S. suis*), and *Pseudomonas aeruginosa* (*P. aeruginosa*) [17,18,19,20]. In addition, the CATH-2-mediated killing of P. aeruginosa significantly inhibits lung inflammation in mice with the reduced recruitment of neutrophils to the lung and inhibition of cytokine and chemokine production compared to heat-killed and gentamycin-killed bacterial infection [20]. However, the exact mechanism of the anti-inflammation of CATH-2 in the host cells is still unclear. We herein reported that CATH-2 performed an anti-inflammatory response independent of bacterial killing. Our study presented that CATH-2 pretreatment attenuated an inflammatory response via the inhibition of the NF-κB/MAPK/NLRP3 signaling pathway by the disruption of lysosome.

Most AMPs exert their anti-inflammatory activity through neutralizing LPS to block inflammatory cytokines’ expression [21]. However, human cathelicidin LL-37 has been reported to exert both pro- and anti-inflammatory effects. For example, LL-37 inhibits an LTA-induced inflammatory effect in mice macrophages by the inhibition of TNF-α and IL-6 production as well as p38 MAPK and Akt phosphorylation [22]. On the contrary, recent studies reported that LL-37 induces skin inflammation in an NLRP3-dependent manner [23]. Furthermore, LL-37 induces the release of TNF-α and IL-1β by activating TLR4/NF-κB/NLRP3 signaling in coronary artery endothelial cells [24]. Our previous study has reported that CATH-2 promotes NLRP3 inflammasome activation and leads to IL-1β secretion in LPS-primed mice macrophages [10]. However, our present study showed that CATH-2 pretreatment attenuated IL-1β and IL-6 secretion in APEC-infected mice macrophages by the inhibition of the NF-κB/MAPK/NLRP3 pathway. Of note, our study showed that CATH-2 inhibited both phosphorylation and protein expression of NF-κB p65 and MAPK (ERK1/2 and JNK) as well as NLRP3 protein expression, indicating that CATH-2 might interact with these proteins, thereby inhibiting their signaling pathway, but the exact interaction mechanism needs to be further studied. However, CATH-2 did not downregulate the ratio of p-ERK/ERK at 60 min post-infection, which is in contrast with the change in p-p65/p65 and p-JNK/JNK. This result indicates that CATH-2 might have a different ability to modulate different signaling pathways. Notably, CATH-2 has been reported to modulate the activation of TLR2 and TLR4 by directly killing bacteria [9]. Our previous study showed that the pretreatment of other AMP CATH-1 could directly inhibit the expression of TLR2 and TLR4 in macrophages [25]. In this study, we pretreated cells with CATH-2, followed by washing steps which excluded directly killing the bacteria, demonstrating that CATH-2 might act on the host, but whether CATH-2 directly acts on TLRs needs to be further studied. These studies demonstrate that CATH-2 exerts a dual role in an inflammatory or anti-inflammatory response dependent on the interaction sequence with the host.

Importantly, the complete activation of NLRP3 inflammasome is required for two signals, including LPS priming, activating NF-κB transcription and the recruitment of ASC activating caspase-1 to process pro-IL-1β into IL-1β. Our results showed that CATH-2 pretreatment inhibited APEC-induced pro-IL-1β expression and caspase-1 activation but did not affect pro-caspase-1 expression, indicating that CATH-2 inhibits both NF-κB activation and NLRP3 inflammasome assembly. However, which of the host factors CATH-2 acts on needs to be further studied.

Lysosomal function plays a key role in the elimination of pathogens. Our previous study demonstrated that CATH-2 induces CTSB leakage in the lysosome in LPS-stimulated macrophages [10], leading to IL-1β release, demonstrating that CATH-2 might induce pyroptosis in LPS-primed macrophages. In addition, human cathelicidin LL-37 has been reported to induce CTSB leakage in the lysosome [26]. Our present study showed that CATH-2 inhibited CTSB mRNA expression and lysosomal acidification, leading to the inhibition of IL-1β. Acidification accelerator ML-SA1 reversed NLRP3 activation inhibited by CATH-2. It has been reported that IL-1β secretion is dependent on CTSB activity [27]. Therefore, we reasonably speculated that CATH-2 disrupted lysosomal function, leading to decreased CTSB activity, thereby attenuating inflammation.

In conclusion, our study revealed that CATH-2 could be a potential lysosomal inhibitor to exhibit its anti-inflammatory effect. Our study increases our knowledge on the anti-inflammatory signaling pathway of animal AMPs.

## 4. Materials and Methods

### 4.1. Animals

The wild-type (WT) C57BL/6 mice were purchased from the Chongqing Academy of Chinese Material Medica (Chongqing, China). All the mice were maintained in Specific Pathogen-Free (SPF) conditions before being used at 8–10 weeks old. All the animal experiments were approved by the Southwest University Ethics Committee, Chongqing, China (IACUC-20210215-07).

### 4.2. Peptides

Chicken cathelicidin-2 CATH-2 (Amino acid sequence: RFGRFLRKIRRFRPKVTITIQGSARF-NH_2_) was synthesized by China Peptides (Shanghai, China) using Fmoc-chemistry and purified by reverse-phase high-performance liquid chromatography to a purity > 95%. Peptides were prepared in distilled water.

### 4.3. Bacterial Strain and Culture

APEC (O78) was kindly provided by Prof. Qingke Kong (College of Veterinary Medicine, Southwest University, Chongqing, China) and cultured in Luria–Bertani Broth (LB) at 37 °C and grown to mid-logarithmic growth phase before testing.

### 4.4. Preparation of Macrophages and APEC Infection

Mice primary peritoneal macrophages were prepared as previously reported [10]. Cells were seeded in 48-well plates or 12-well plates and incubated at 37 °C with 5% CO_2_. After 2 h of incubation, CATH-2 (2.5 μM) was added to cells for 6 h and then washed away, followed by bacterial infection at a multiplicity of infection (MOI) of 5 for 1 or 2 h. Subsequently, gentamycin (100 μg/mL) was added to cells to kill extracellular bacteria, and the cells continued to incubate for the indicated time for the assays described below. To promote lysosomal acidification, ML-SA1 (20 μM) was added 24 h prior to the CATH-2 treatment.

### 4.5. Enzyme-Linked Immunosorbent Assay (ELISA)

Cells in 48-well plates were pretreated with CATH-2 followed by bacterial infection. After 24 h of infection, the supernatants and cell lysates were collected to determine the concentration of cytokines, including IL-1β, IL-1α, IL-6, and IL-12 (Invitrogen, Carlsbad, CA, USA), using the ELISA according to the manufacturer’s instructions.

### 4.6. Cell Viability

Cells were treated with CATH-2 (2.5 μM) for 6 h. After incubation, peptides were washed away and continued to incubate for an additional 24 h. Then, cell medium was replaced by 10% WST reagent (Biomed, Beijing, China). Finally, the same volume of supernatants was transferred in a new 96-well plate, and absorbance was measured at 450 nm with a microplate reader (Bio-Rad, Tokyo, Japan).

### 4.7. Quantitative Real-Time Polymerase Chain Reaction (RT-qPCR)

Cells in 12-well plates were pretreated with CATH-2 followed by bacterial infection. After 6 h of infection, total RNA was extracted by TRIzol Reagent (Life Technologies, Carlsbad, CA, USA). Then, RNA (1 μg) was reverse-transcribed by using the PrimeScript^®^ RT reagent Kit (Takara, Beijing, China). Subsequently, the cDNA was subjected to qPCR using TB 765 Green^®^ Premix Ex Taq™ (Takara, Beijing, China). Finally, qPCR was performed using the CFX96 (Bio-Rad, Hercules, CA, USA). Primers were used as follows: IL-1β forward 5′-GAA ATG CCA CCT TTT GAC AGT G-3′ and reverse 5′-TGG ATG CTC TCA TCA GGA CAG-3′, IL-6 forward 5′-TTC CAT CCA GTT GCC TTC TTG-3′ and reverse 5′-GAA GGC CGT GGT TGT CACC-3′, cathepsin B (CTSB) forward 5′-GGC TCT TGT TGG GCA TTT GG-3′and reverse 5′-ACT CGG CCA TTG GTG TGA AT-3′, β-actin forward 5′-TGG AAT CCT GTG GCA TCC ATG AAA C-3′ and reverse 5′-TAA AAC GCA GCT CAG TAA CAG TCC G-3′. Gene expression was represented via gene normalization against β-actin expression.

### 4.8. Western Blot Analysis

After infection at the indicated time, supernatants and cell lysates using 1 × SDS loading buffer (Biosharp, Chongqing, China) were collected, followed by the performance of SDS-PAGE. Next, they were transferred onto a polyvinylidene difluoride (PVDF) membrane by electroblotting. Subsequently, the membranes were blocked with 5% nonfat dry milk and then immunoblotted with the indicated antibodies (Abs) including anti-IL-1β (R&D, Minneapolis, MN, USA), anti-caspase-1 p20 (AdipoGen, San Diego, CA, USA), anti-pro-IL-1β, anti-pro-caspase-1, anti-NF-κB p65, anti-NF-κB p-p65, anti-ERK, anti-p-ERK, anti-JNK, anti-p-JNK, and anti-β-actin (Beyotime, Beijing, China). Finally, protein bands were detected by ECL detection reagent (Biosharp, Chongqing, China).

### 4.9. Immunofluorescence Staining

After 2 h of infection, cells were fixed in 4% paraformaldehyde (Sango Biotech, Shanghai, China) for 30 min and then permeabilized with 0.1% Triton X-100 in PBS for 10 min. Subsequently, cells were blocked with 5% Bovine Serum Albumin (BSA) in PBS for 1 h. After blocking, cells were incubated with primary Abs containing anti-NLRP3 (Bioss, Beijing, China) and anti-ASC (Santa Cruz, CA, USA) for 12 h at 4 °C. After washing, cells were incubated with second Abs including goat anti-mouse IgG (H&L) Alexa fluor 488 and goat anti-rabbit IgG (H&L) Alexa fluor 594 (Abcam, London, UK) for 1 h at RT. DAPI was used to stain cell nuclei. Finally, cells were maintained in antifading medium and observed using the fluorescence microscopy (Olympus, Tokyo, Japan).

### 4.10. Detection of Lysosomal Leakage and Acidification

Cells were loaded with Alexa 488 Dextran (20 μM), 10 kDa (Life Technologies) for 1 h prior to CATH-2 treatment and bacterial infection for a further 3 and 6 h. For the detection of lysosomal acidification, after 2 h of infection, LysoSensor Green DND-189 (3 μM) (Yeasen, Shanghai, China) was added for 1 h. After incubation, the cell culture medium was replaced by fresh medium, and cells were observed using fluorescence microscopy (Olympus, Tokyo, Japan).

### 4.11. Statistical Analysis

Data are shown as mean ± SEM. Each experiment was repeated three times, and each group has three samples. A one-way ANOVA was used to analyze statistical significance among the different groups. The *p* value showed statistical significance. * *p* < 0.05, ** *p* < 0.01, *** *p* < 0.001, ns = no significance.

## Data Availability

All the data generated for this study are included in the article.

## Supplementary Materials

The following supporting information can be downloaded at: https://www.mdpi.com/article/10.3390/ijms252312572/s1.
