# Supplementary figures and images for: Antimicrobial Peptide CATH-2 Attenuates Avian Pathogenic E. coli-Induced Inflammatory Response via NF-κB/NLRP3/MAPK Pathway and Lysosomal Dysfunction in Macrophages
Source: Int J Mol Sci. 2024 Nov 22;25(23):12572. doi: 10.3390/ijms252312572 (PMC11641483; doi:10.3390/ijms252312572)

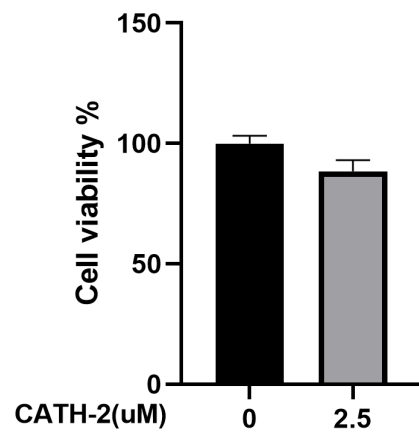

**Supplementary Figure S1.** Cell viability. Cell viability was shown under the treatment of CATH-2.

Supplement: Supplementary file 1 [file ijms-25-12572-s001.zip › ijms-3313903-supplementary.pdf]
